# Nutritional status associated with physical activity in active older adults in southern Ecuador

**DOI:** 10.3389/fragi.2026.1723742

**Published:** 2026-03-18

**Authors:** Lorena Encalada-Torres, Ivanna Buri-Encalada, Victoria Abril-Ulloa, Christian Rodas-Guamán, María Quito-Parra

**Affiliations:** Research Group on Public Health, Nutrition, and Physical Activity in the Life Cycle, Faculty of Medical Sciences, University of Cuenca, Cuenca, Ecuador

**Keywords:** IPAQ, mini nutritional assessment, nutritional status, older adult, physical activity

## Abstract

**Introduction:**

The increase in the number of older adults at a global level is increasing the incidence of morbidity and mortality associated with lack of physical activity and malnutrition in this vulnerable group. The objective was to determine the nutritional status and its association with the level of physical activity in active older adults in southern Ecuador.

**Methods:**

Analytical cross-sectional study in 400 older adults, performing anthropometry, and applying physical activity questionnaires (IPAQ-c) and Mini Nutritional Assessment (MNA) prior to signing the informed consent. The data were analyzed in the SPSS v.15.0 program, using descriptive statistics, OR with 95% CI and chi square with its p value <0.05.

**Results:**

Malnutrition according to BMI was 59.6%, with overweight predominating (30.8%); 33.2% had low physical activity; there was a significant association between malnutrition and low physical activity with men (p = 0.013); obesity associated with low physical activity and age (p = 0.028), men (p = 0.029), low education (p = 0.029); according to MNA malnutrition was 39.5%, of these 42.4% presented low physical activity; there was an association between malnutrition and low physical activity in ≥85 years, men, with a partner, low education and rural residents (p = 0.000).

**Conclusion:**

The prevalence of malnutrition in older adults is increasing and is associated with low physical activity, being a public health problem that requires a comprehensive intervention by health authorities for a better quality of life of older adults.

## Introduction

The life expectancy of the world’s population has increased, causing rapid population aging (Abd et al., 2017). The World Health Organization (WHO) estimates that by 2050 the number of older adults will exceed 2 billion (22%), doubling the 1.1 billion in 2015 (World Health Organization, 2024b). In the Americas, the older adults’ population reached 13.4% of the total population in 2022. It is estimated that by 2030 their proportion will rise to 16.5% and by 2050 to 25.1% (Economic Commission for Latin America and the Caribbean, 2022). In Ecuador, life expectancy has increased to 77.4 years by 2024, projecting an increase from 1,317,749 older adults in 2024 to 1,868,583 older adults in 2030 (INEC, 2024). Therefore, the increase in the population over 65 years of age will increase the prevalence of chronic non-communicable diseases (CNCDs) and will require significant investments in public health for the care of older adults (World Health Organization, 2024a).

In this context, maintaining good nutritional status and engaging in physical activity are essential for healthy aging. Optimal nutrition is crucial for maintaining functional capacity and independence in aging (Sugiura et al., 2016). On the other hand, malnutrition is characterized by a decrease in food intake or absorption, leading to deficiencies in essential nutrients, weakness, and reduced muscle strength (Sugiura et al., 2016; Wang et al., 2024; Rajabi et al., 2021; Lorés et al., 2020), causing inability in older adults to perform simple activities, an increased risk of falls, and decreased physical activity levels (World Health Organization, 2023; Füzéki et al., 2017).

For the study of nutritional status together with anthropometric measurements, the Mini Nutritional Assessment (MNA) has shown that it can predict the risk of malnutrition even before any changes in the body such as weight loss occur (Abd et al., 2017; Rajabi et al., 2021; Guerrero-García et al., 2016), it is easy to use and feasible in any clinical care setting, with a sensitivity of 96%, specificity of 98% and predictive value of 97% (Otero and Rosas, 2017). However, in several older adults’ studies with MNA the results vary by region. Thus, a Spanish study by Montejano et al., established that 26.5% were at risk of malnutrition and 0.9% were undernourished (Montejano et al., 2017); in Japan, Sugiura et al., showed 16.8% at high risk of malnutrition with difficulty in performing daily activities (Sugiura et al., 2016). In Latin America, the prevalence of malnutrition is higher, according to the study carried out by Zukeran et al., in Brazil, 3.1% presented malnutrition, being directly proportional to the loss of functional capacity (Zukeran et al., 2019); while in Colombia, the study conducted by Giraldo-Giraldo et al., showed that 41.4% were at risk of malnutrition, and 5.4%, undernutrition in the older adults population (Giraldo-Giraldo et al., 2023), and Otero M, Rosas G, showed that 60.1% were at risk of malnutrition and 8.1% were malnourished (Otero and Rosas, 2017), increasing the prevalence in countries such as India; in which, a study by Banerjee et al., showed that 70% of older adults were at risk of malnutrition and 19.5% were malnourished (Banerjee et al., 2018).

Some studies, including a systematic review showed healthy dietary patterns were associated with better quality of life in one or more domains (Govindaraju et al., 2018). Another study found that the prevalence of malnutrition risk was 34.0% in institutionalized older adults and 28.1% in older adults living in the community, according to the MNA; showing that BMI and muscle mass were significantly associated with different components of the SF-36 quality of life scale in older adults, concluding that periodic nutritional assessment and nutritional interventions in older adults are essential to prevent malnutrition-related comorbidity and improve quality of life (Kanbur-Usuğ et al., 2025). This is supported by other studies in Brazil that have found a significant association between quality of life and nutritional status (DamiÃ£o et al., 2018).

The WHO establishes that any action performed by a musculoskeletal system that results in energy expenditure is considered physical activity (World Health Organization, 2023). The term includes not only physical exercise, which is a planned and repetitive activity, but also other activities of daily living, at work or at home, and recreational activities such as outdoor activities, gardening, walking, cycling, and active transportation (Litt et al., 2023). However, physical activity primarily focuses on leisure activities, commuting (walking, cycling), housework, play, work activities (if the person involved is economically active), or exercise and family activities (Lorés et al., 2020; World Health Organization, 2023; Litt et al., 2023).

On the other hand, physical inactivity is an important risk factor for NCDs (World Health Organization, 2023; Cao et al., 2022; López et al., 2022), and can double the risk of death from older adults (Litt et al., 2023), representing a significant global health burden. It is attributed to more than 7% of deaths from all causes and cardiovascular diseases; and 8% of NCDs; and is more prevalent in low-income countries (Katzmarzyk et al., 2022); despite this, a high proportion of older adults do not meet the WHO minimum physical activity recommendations (Steene-Johannessen et al., 2016). It has been established that public expenditure generated by the treatment of NCDs is high, which can be prevented by practicing physical activity. A report from the U.S. Centers for Disease Control and Prevention (CDC) estimates that investments of one dollar to promote moderate physical activity provide savings of US$3.20 in future medical expenses (Crespo-Salgado et al., 2015).

In Chile, a study by Barrón et al. showed that 17% of the older adults’ population was sedentary (Barrón et al., 2017); while the study by Chimbo et al. determined that 18% of older adults had a low level of physical activity or were inactive (Chimbo et al., 2016). The study by Mejía et al. in Peru determined that 67.8% of older adults did not engage in physical activity and linked this inactivity with a larger waist circumference and NCDs (Mejia et al., 2017). On the other hand, a study by Pandey A in the United States suggests that the Rehabilitation Therapy in Older Acute Heart Failure Patients (REHAB-HF) intervention may be effective among frail older adults with heart failure who have a high burden of functional impairment (Pandey et al., 2023).

For research on physical activity levels in the population, the most commonly used assessment method is retrospective questionnaires (Rääsk et al., 2017), thanks to their ability to assess levels in large populations across different countries, their ease of use, and their low cost. The most widely used is the International Physical Activity Questionnaire (IPAQ). This has been validated and widely adopted in large studies such as the European Commission’s Eurobarometer and the WHO’s World Health Survey (Steene-Johannessen et al., 2016), as well as in research on older adults (López et al., 2022).

In this context, while evidence exists in Ecuador regarding different levels of physical activity and their influence on nutritional status, most studies are based on younger adult populations or do not adequately distinguish between fat mass and muscle mass. This can lead to misinterpretations in older adults due to sarcopenia and the physiological changes associated with aging. Furthermore, a gap persists related to the lack of research evaluating variables such as intensity, frequency, and type of exercise (aerobic, strength, balance), which have differentiated influences on nutritional status and clinically relevant outcomes, such as mobility, risk of falls, and quality of life. This lack of specific evidence limits the design of effective and tailored interventions for individuals over 65, especially within public health systems in developing countries like Ecuador and in populations with multiple comorbidities, such as older adults.

It is necessary to begin by developing regional research that demonstrates the reality of their own vulnerable populations, such as the elderly group, before being able to start with intervention programs to improve their quality of life. For these reasons, the objective of this research was to determine nutritional status and its association with physical activity levels in active older adults in Southern Ecuador. For these reasons, the objective of this research was to determine nutritional status and its association with physical activity levels in active older adults in Southern Ecuador.

## Materials and methods

### Type of study and sample

This was a cross-sectional analytical study of 400 older adults in the province of Azuay, southern Ecuador, weighted according to the population of urban and rural areas in the different parishes. The EPIDAT v3.1 program was used to obtain the sample with the following sampling restrictions: Population: 55,834 older adults from both urban and rural parishes in the province of Azuay, expected proportion: 15.8% (with physical activity as an associated factor) (37), error: 5%, with a 95% confidence interval (95% CI), obtaining a total sample of 204, to which a 10% possible non-response was added, with a minimum size of 225 older adults, resulting in a final population of 400 older adults. The sample was weighted according to the population of the urban and rural areas of the different parishes.

The inclusion criteria for this study included both men and women aged 65 years or older who had lived in the province of Azuay for at least 1 year and who signed informed consent. While, older adults with various disabilities (cognitive, hearing, or verbal), psychiatric illnesses, and/or altered states of consciousness were excluded.

### Instruments and procedure

For data collection, the following procedures were carried out beforehand: 1) identification of the different cantons of the province of Azuay and preparation of their respective geographic map; 2) division of the geographic area into parishes, sectors and addresses (for rural areas), identifying the census zones according to the INEC; 3) simple random selection using the program https://www.graphpad.com/quickcalcs/randmenu/ of the parishes, sectors and addresses; if the AM was not found at their address or did not wish to participate, the next address was selected in a clockwise direction, until the sample was completed in each geographic area.

Demographic data were collected through a structured survey developed by the authors. This survey included information on age (in completed years at the time of the survey), sex (male and female), usual residence (urban or rural), marital status (verified by national identity card), previous occupation (classified as manual or non-manual labor), and education level (considering the academic regulations of the Ecuadorian education system for older adults). Anthropometry was performed to determine the nutritional status of the older adults using a Seca 700 scale with a height rod. This scale has a capacity of up to 220 kg and a kilogram-to-pound conversion. Its measuring range is 60–200 cm, allowing for weighing and measuring in a single operation. The scale is equipped with transport wheels for flexible and mobile use. Prior to use, it was calibrated according to WHO international metrology standards.

For the calibration of the scale (weight), certified standard weights (class M1 or higher according to OIML) were used. These were placed in the center of the platform, and three load points were verified: low load (5 kg), medium load (≈50% of capacity), and high load (≈80–100% of capacity). The displayed value was checked against the standard value, allowing for an error of ±100 g. For the calibration of the height rod, a certified metal ruler was used, and measurements were verified at least two points: low point (100 cm) and high point (170 cm). It was confirmed that the height rod was perpendicular to the ground and that the sliding head was at a right angle to the scale, with an acceptable error of ≤ ±0.5 cm (CDC, 2020).

For weight measurement, the scale was placed on a firm, flat, and level surface. The scale was checked to ensure it was zeroed before use, and the calibration screw was adjusted as needed. The cursors (movable weights) were set to 0 kg, and the indicator needle was confirmed to be aligned with the balance mark.

For weight, the patient was placed centrally and symmetrically on the platform, wearing minimal clothing and barefoot. To measure weight, first move the large cursor (lower bar) until it is close to the estimated weight, then adjust with the small cursor (upper bar) until the needle is exactly centered over the balance point. Ensure the patient does not move or lean during the measurement and only read the weight when the needle is stable.

For height, the patient was placed standing, without headgear, arms suspended freely at the sides, barefoot, standing with heels together, head, buttocks, and heels in the Frankfort plane, attached to the height rod. Next, we ask the patient to take a deep breath and then maintain an upright posture. We gently lower the head of the height rod until it makes firm contact with the top of the skull, ensuring that it is perpendicular to the s height rod column, avoiding compressing the scalp. We confirm that the patient remains still and looks straight ahead, and then we proceed to read the height on the scale.

Body mass index (BMI) was calculated using a simple mathematical formula that relates body weight to height (BMI = weight in kg/height in meters squared). Then, the WHO qualitative-quantitative scale for older adults was used, which defines obesity as ≥ 32; overweight as ≥ 28 to <32; normal weight as > 23 to <28; and underweight as ≤ 23 (Urgessa, 2022). Malnutrition was defined as the sum of underweight, overweight, and obesity.

Malnutrition is not limited to underweight; it encompasses overweight and obesity, which lead to excess malnutrition due to an abnormal accumulation of fat resulting from energy intake exceeding expenditure. BMI is the standard population indicator used by the WHO to classify this imbalance, which poses a health risk to older adults and is associated with chronic diseases such as diabetes, hypertension, and some types of cancer (World Health Organization, 2024b). Excess malnutrition represents a dysfunctional state of adipose tissue, which transforms from an energy reserve into an active inflammatory tissue, from an aesthetic concern to a complex metabolic disease. BMI is a direct marker of this form of malnutrition, reflecting the excess fat mass that impairs health (Ravasco et al., 2010). Furthermore, many low- and middle-income countries face the double burden of malnutrition. These countries struggle with problems related to infectious diseases and malnutrition, and risk factors for chronic non-communicable diseases such as obesity and overweight (Saxena et al., 2025).

For abdominal circumference, a dry-brand measuring tape with 1 mm precision was used. The participant was positioned on a flat surface with their torso uncovered and in an upright position. The upper edge of the iliac crest and the lower edge of the last rib were palpated to determine the average distance between these two points. The measuring tape was then placed horizontally around the abdomen, adjusted without compressing the skin, ensuring that the tape was parallel to the floor along the entire abdominal circumference after the participant exhaled. Reference values of ≤90 cm for men and ≤80 cm for women were considered; values above these were considered visceral obesity (Kunstmann, 2008). Two measurements of height, weight, and abdominal circumference were taken, and the averages were calculated.

In addition, the Mini Nutritional Assessment (MNA) questionnaire (Urgessa, 2022) was administered. The MNA consists of simple measurements and brief questions that can be completed in approximately 10 min. It comprises 18 questions divided into four areas: lifestyle, medication, physical and mental status, dietary history (including questions about daily dietary intake and eating problems), and self-perceived health. Anthropometric measurements were also taken, including BMI, arm and thigh circumference, and weight loss. The sum of the MNA questionnaire score distinguishes between elderly patients with: 1) adequate nutritional status (MNA ≥24); 2) risk of malnutrition (MNA between 17 and 23.5); and 3) protein-energy malnutrition (MNA <17). There are several studies with the MNA questionnaire and older adults that have shown since its application in older adults a sensitivity of 96%, a specificity of 98% and a predictive value of 97%, to establish the risk of malnutrition (Otero and Rosas, 2017).

For physical activity, the IPAQ-c questionnaire (López et al., 2022) was used, which assessed physical activity over the past 7 days, recording the time a person spends sitting or lying down, work activities, physical-sports activities, transportation (walking), and the intensity of the activities: moderate intensity (cycling) and vigorous intensity (running or aerobic exercises) (Litt et al., 2023). It contains seven questions that measure: frequency (days/week), duration (minutes/day) of participation in vigorous and moderate intensity activities and walking in 10-min episodes. To determine the total level of physical activity, the scores for each activity are calculated in METs (Metabolic Equivalents) minutes per week. This is obtained by multiplying the MET intensity of each activity (Walking: 3.3 METs; moderate physical activity: 4 METs; and vigorous physical activity: 8 METs) by the minutes spent on that activity in a day and by the number of days per week it is performed. One MET represents the energy expended while sitting at rest and is equivalent to 3.5 mL O2/Kg/min (Steene-Johannessen et al., 2016).

### Statistical analysis

The data were tabulated and analyzed using SPSS version 15.0. Descriptive statistics such as frequencies and percentages were used for categorical variables, while for continuous variables, measures of central tendency such as the mean and measures of dispersion such as the standard deviation (SD) were used. To assess whether the distribution of nutritional status (categorical variables BMI, MNA) differs according to the level of physical activity (low/moderate/high), and to identify statistical significance, Pearson’s Chi-square test was used with a p-value <0.05. To quantify the strength of the association between the exposure measurement (level of physical activity) and the event (malnutrition), expressing how many times greater the probability of malnutrition is in people with low physical activity compared to those with moderate or high activity, the Odds Ratio with its 95% confidence interval was used.

The study was authorized by the Bioethics Committee of the Health Department of the University of Cuenca (COBIASUCuenca) (protocol code 2018-059EO).

## Results

Of the study population, the prevalent age groups were 65–74 years and 75–84 years, with the average age being 77.17 (±7.7) years. The majority were women (60.2%). Their marital status was married (49.5%), with 95% self-identifying as mestizo and with incomplete primary education. A quarter of the study population were small-scale agricultural workers in their previous occupation, and 53% were from the rural sector (Table 1).

**TABLE 1:** Demographic characteristics of the study population.

Demographic variables	n = 400	100%
^a^Age		
65 – 74	161	40.3
75 – 84	161	40.3
≥85	78	19.5
Sex		
Woman	241	60.2
Man	159	39.8
Marital status		
Single	43	10.8
Married	198	49.5
Widowed	127	31.8
Divorced	24	6.0
Common-law union	8	2.0
Level of education		
No education	47	11.8
Incomplete primary education	144	36.0
Complete primary education	122	30.5
Incomplete secondary education	27	6.8
Complete secondary education	23	5.8
Incomplete higher education	12	3.0
Complete higher education	21	5.3
Other	4	1.0
Previous occupation		
Small agricultural worker	104	26.0
Domestic chores	80	20.0
Artisan and small industrialist	61	15.3
Private employee	43	10.8
Small trader	40	10.0
Public employee	29	7.2
Laborer	19	4.8
Independent professional	19	4.8
Other	3	0.8
Senior merchant	2	0.5
Residence		
Urban	188	47.0
Rural	212	53.0

^a^ Mean: 77.17; Standard Deviation (SD): ±7.7.

One-third of the elderly were overweight, and 17% of them lived in rural areas; 92.5% of women and 83.6% of men had abdominal obesity, and most of them lived in rural areas (Table 2).

**TABLE 2:** Anthropometric data and geographic location of the study population.

Demographic variables	Geographic location					
	Urban		Rural		Total	
​	n = 188	47%	n = 212	53%	n = 400	100%
^a^BMI						
Thinness	13	3.3	28	7.0	41	10.3
Normal	82	20.5	80	20.0	162	40.5
Overweight	55	13.8	68	17.0	123	30.8
Obesity	38	9.5	36	9.0	74	18.5
^b^Abdominal perimeter women						
Abdominal obesity (≥80 cm)	106	44.0	117	48.5	223	92.5
Normal (<80 cm)	7	2.9	11	4.6	18	7.5
^c^Abdominal perimeter men						
Abdominal obesity (≥90 cm)	66	41.5	67	42.1	133	8.6
Normal (<90 cm)	9	5.7	17	10.7	26	16.4

^a^ BMI: Mean 28.12 (±4.27).

^b^ Abdominal perimeter women: Mean 95.25 (±11.84).

^c^ Abdominal perimeter men: Mean 99.24 (±10.72).

In 36.0% of older adults, according to the MNA, they were at risk of malnutrition with a higher prevalence among those aged 75–84 years (43.7%), women (72.2%), married (40.9%), incomplete primary education (48.6%), from the rural sector (63.9%) and manual occupation (80.6%); while malnutrition was evident in both young older adults and the so-called long-lived older adult (35.7%), female (78.6%), widowed (64.3%), incomplete primary education (42.8%), from rural areas (78.6%) and manual occupation (85.7%) (Table 3).

**TABLE 3:** Malnutrition according to MNA and demographic characteristics.

Demographic variables	MNA							
	Satisfying		Risk of malnutrition		Malnutrition		Total	
​	n	%	n	%	n	%	n	%
Total population	242	60.5	144	36.0	14	3.5	400	100.0
Age								
65–74	114	47.1	42	29.2	5	35.7	161	40.3
75–84	94	38.8	63	43.7	4	28.6	161	40.3
>85	34	14.1	39	27.1	5	35.7	78	19.5
Sex								
Female	126	52.1	104	72.2	11	78.6	241	60.3
Male	116	47.9	40	27.8	3	21.4	159	39.7
Marital status								
Single	24	10.0	16	11.1	3	21.4	43	10.7
Married	137	56.6	59	40.9	2	14.3	198	49.5
Widowed	62	25.6	56	38.9	9	64.3	127	31.8
Divorced	16	6.6	8	5.6	0	0.0	24	6.0
Common-law marriage	3	1.2	5	3.5	0	0.0	8	2.0
Education								
No education	24	10.0	19	13.2	4	28.6	47	11.7
Incomplete primary education	68	28.1	70	48.6	6	42.8	144	36.0
Complete primary education	74	30.6	44	30.5	4	28.6	122	30.5
Incomplete secondary education	22	9.1	5	3.5	0	0.0	27	6.8
Complete secondary education	23	9.5	0	0.0	0	0.0	23	5.8
Incomplete higher education	9	3.7	3	2.1	0	0.0	12	3.0
Complete higher education	18	7.4	3	2.1	0	0.0	21	5.2
Other	4	1.6	0	0.0	0	0.0	4	1.0
Place of residence								
Rural	109	45.0	92	63.9	11	78.6	212	53.0
Urban	133	55.0	52	36.1	3	21.4	188	47.0
Occupation								
Manual	136	56.2	116	80.6	12	85.7	264	66.0
Non-manual	106	43.8	28	19.4	2	14.3	136	34.0

Regarding the level of physical activity, a large part of the older adults studied (43.3%) were found to be at a moderate level, followed by a low inactive level (29.3%); of which, the moderate level of physical activity was more frequent in the young older adult (49.1%), in women (59.5%), in married people (51.4%), in older adults with incomplete primary education (29.5%) and in urban areas (51.4%); while the low inactive level of physical activity was found more in older adults between 75 and 84 years of age (41.9%), in women (58.1%), in widows (47.9%), older adults with incomplete primary education (41.0%), belonging to the rural sector (41.0%) and in manual occupation (67.5%) (Table 4).

**TABLE 4:** Physical activity level (IPAQ-short) and demographic characteristics.

​	Physical activity level (IPAQ- short)							
	High		Moderate		Low or inactive		Total	
Demographic variables	n	%	n	%	n	%	n	%
Total population	110	27.5	173	43.3	117	29.3	400	100.0
Age								
65–74	49	44.5	85	49.1	27	23.1	161	40.3
75–84	51	46.6	61	35.3	49	41.9	161	40.3
>85	10	9.1	27	15.6	41	35.0	78	19.5
Sex								
Female	70	63.6	103	59.5	68	58.1	241	60.3
Male	40	36.4	70	40.5	49	41.7	159	39.7
Marital status								
Single	11	10.0	23	13.3	9	7.7	43	10.7
Married	64	58.2	89	51.4	45	38.5	198	49.5
Widowed	27	24.5	44	25.4	56	47.9	127	31.8
Divorced	6	5.5	14	8.1	4	3.4	24	6.0
Common-law marriage	2	1.8	3	1.7	3	2.6	8	2.0
Education								
No education	17	15.5	19	11.0	11	9.4	47	11.7
Incomplete primary education	45	40.9	51	29.5	48	41.0	144	36.0
Complete primary education	37	33.6	48	27.7	37	31.6	122	30.5
Incomplete secondary education	4	3.6	17	9.8	6	5.1	27	6.8
Complete secondary education	2	1.8	15	8.7	6	5.1	23	5.8
Incomplete higher education	3	2.7	5	2.9	4	3.4	12	3.0
Complete higher education	1	0.9	15	8.7	5	4.3	21	5.2
Other	1	0.9	3	1.7	0	0.0	4	1.0
Place of residence								
Rural	80	72.7	84	48.6	48	41.0	212	53.0
Urban	30	27.3	89	51.4	69	59.0	213	47.0
Occupation								
Manual	82	74.5	103	59.5	79	67.5	264	66.0
Non-manual	28	25.5	70	40.5	38	32.5	136	34.0

According to the MNA scale and physical activity level, more than half of the population (57.1%) of older adults with malnutrition had low or inactive physical activity levels. Similarly, the majority of older adults at risk for malnutrition (41.0%) had low or inactive physical activity levels. On the other hand, the population with satisfactory nutritional status (51.7%) had moderate physical activity levels (Table 5).

**TABLE 5:** MNA and physical activity level (IPAQ-short).

Physical activity level (IPAQ-short)	MNA							
	Malnutrition		Risk of malnutrition		Satisfactory nutritional status		Total	
​	n = 14	100.0%	n = 144	100.0%	n = 242	100.0%	n = 400	100%
Low	8	57.1	59	41.1	50	20.7	117	29.25
Moderate	2	14.3	46	31.9	125	51.7	173	43.25
High	4	28.6	39	27.1	67	27.7	110	27.5

Low or inactive physical activity levels were statistically significantly associated with malnutrition based on BMI (OR 1.202; 95% CI 1.022–1.414; p = 0.036) and MNA (OR 2.827; 95% CI 1.815–4.404; p = 0.000); and within the BMI classification with obesity (OR 1.51; 95% CI 1.036–2.204; p = 0.038) (Table 6).

**TABLE 6:** Relationship between nutritional status according to BMI and MNA and the level of physical activity.

Physical activity level low or inactive	BMI						
	^a^Malnutrition		Normal		OR	CI 95%	*p*
​	n = 238	100%	n = 162	100%	​	​	​
Yes	79	33.2	38	23.5	1.202	1.022–1.414	0.036
No	159	66.8	124	76.5			
​	Thinness		Normal		​	​	​
​	n = 41	100%	n = 162	100%	​	​	​
Yes	13	31.7	38	23.5	1.384	0.778–2.462	0.277
No	28	68.3	124	76.5			
​	Overweight		Normal		​	​	​
​	n = 123	100%	n = 162	100%	​	​	​
Yes	39	31.7	38	23.5	1.254	0.952–1.652	0.12
No	84	68.3	124	76.5			
​	Obesity		Normal		​	​	​
​	n = 74	100%	n = 162	100%	​	​	​
Yes	27	36.5	38	23.5	1.511	1.036–2.204	0.038
No	47	63.5	124	76.5			

Physical activity level low or inactive	MNA						
	^b^Malnutrition		Normal		OR	CI 95%	*P*
​	n = 158	100%	n = 242	100%	​	​	​
Yes	67	42.4	50	20.7	2.827	1.815–4.404	0.000
No	91	57.6	192	79.3			

^a^ Malnutrition corresponds to the sum of thinness, overweight and obesity.

^b^ Malnutrition corresponds to the sum of: malnutrition and risk of malnutrition.

The factors that showed a statistically significant association with malnutrition and having low or inactive physical activity were: being male (OR 2.438; 95% CI 1.194–4.975; p = 0.013) and having a manual labor occupation (OR 2.297; 95% CI 1.290–4.090; p = 0.004) (Table 7).

**TABLE 7:** Relationship between malnutrition (BMI) and level of physical activity (IPAQ-Short), according to demographic variables.

​	​	Malnutrition (BMI)						
Demographic variables	Low or inactive physical activity	Yes		No		​	​	​
​	​	n = 238	100%	n = 162	100%	OR	CI 95%	*p*
Age								
65–74	Yes	20	12.4	7	4.3	2.18	0.864–5.505	0.094
	No	76	47.2	58	36			
75–84	Yes	32	19.9	17	10.6	1.518	0.757–3.046	0.239
	No	62	38.5	50	31.1			
≥85	Yes	27	34.6	14	17.9	1.469	0.588–3.674	0.41
	No	21	26.9	16	20.5			
Sex								
Woman	Yes	45	18.7	23	9.5	1.237	0.687–2.227	0.479
	No	106	44	67	27.8			
Man	Yes	34	21.4	15	9.4	2.438	1.194–4.975	0.013
	No	53	33.3	57	35.8			
Marital status								
Single	Yes	47	24.2	22	11.3	1.625	0.876–3.014	0.122
	No	71	36.6	54	27.8			
Partnered	Yes	32	15.5	16	7.8	1.591	0.808–3.132	0.177
	No	88	42.7	70	34			
Educational level								
Low	Yes	65	20.8	31	9.9	1.573	0.949–2.606	0.078
	No	124	39.6	93	29.7			
High	Yes	14	16.1	7	8	1.771	0.634–4.952	0.272
	No	35	40.2	31	35.6			
Residence								
Rural	Yes	34	16	14	6.6	1.636	0.815–3.281	0.164
	No	98	46.2	66	31.1			
Urban	Yes	45	23.9	24	12.8	1.783	0.967–3.288	0.063
	No	61	32.4	58	30.9			
Job occupation								
Manual labor	Yes	58	22	21	8	2.297	1.290–4.090	0.004
	No	101	38.3	84	31.8			
Non-manual labor	Yes	21	15.4	17	12.5	0.852	0.400–1.814	0.678
	No	58	42.6	40	29.4			

No factors were found to be statistically significantly associated with BMI-related leanness, low physical activity, or inactivity. The only factor that showed a statistically significant association between overweight and low or inactive physical activity was manual labor (OR 2.196; 95% CI 1.130–4.267; p = 0.019). The factors that showed a statistically significant association with obesity and having low or inactive physical activity were: being between 75 and 84 years of age (OR 2.614; 95% CI 1.096–6.238; p = 0.028), male sex (OR 3.109; 95% CI 1.090–8.872; p = 0.029), low educational level (OR 2.091; 95% CI 1.070–4.085; p = 0.029) and manual labor occupation (OR 3.111; 95% CI 1.478–6.548; p = 0.002) (Table 8).

**TABLE 8:** Relationship between obesity (BMI) and level of physical activity (IPAQ-short), according to demographic variables.

​	​	Obesity						
Demographic variables	Low or inactive physical activity	Yes		No		​	​	​
		n = 74	100%	n = 162	100%	OR	CI 95%	*p*
Age								
65–74	Yes	6	6.2	7	7.2	1.912	0.585–6.251	0.278
	No	26	26.8	58	59.8			
75–84	Yes	16	15.8	17	16.8	2.614	1.096–6.238	0.028
	No	18	17.8	50	49.5			
≥85	Yes	5	13.2	14	36.8	1.905	0.384–9.444	0.426
	No	3	7.9	16	42.1			
Sex								
Woman	Yes	18	12.5	23	16.0	1.457	0.696–3.046	0.317
	No	36	25.0	67	46.5			
Man	Yes	9	9.8	15	16.3	3.109	1.090–8.872	0.029
	No	11	12.0	57	62			
Marital status								
Single	Yes	16	14.0	22	19.3	1.785	0.792–4.024	0.160
	No	22	19.3	54	47.4			
Partnered	Yes	11	9.0	16	13.1	1.925	0.788–4.703	0.147
	No	25	20.5	70	57.4			
Educational level								
Low	Yes	23	12.8	31	17.2	2.091	1.070–4.085	0.029
	No	33	18.3	93	51.7			
High	Yes	4	7.1	7	12.5	1.265	0.318–5.035	0.738
	No	14	25.0	31	55.4			
Residence								
Rural	Yes	10	8.6	14	12.1	1.813	0.716–4.595	0.206
	No	26	22.4	66	56.9			
Urban	Yes	17	14.2	24	20	1.956	0.882–4.341	0.097
	No	21	17.5	58	48.3			
Job occupation								
Manual labor	Yes	21	13.7	21	13.7	3.111	1.478–6.548	0.002
	No	27	17.6	84	54.9			
Non-manual labor	Yes	6	7.2	17	20.5	0.706	0.241–2.067	0.524
	No	20	24.1	40	48.2			

The factors that showed a statistically significant association with malnutrition (MNA) and having low or inactive physical activity were: ≥85 years (OR 5.723; 95% CI 2.144–15.274; p = 0.000), male (OR 4.598; 95% CI 2.178-9.705; p = 0.000), with a partner (OR 3.486; 95% CI 1.1781–6.824; p = 0.000), having a low level of education (OR 2.655; 95% CI 1.616–4.363; p = 0.000), being a rural resident (OR 4.994; 95% CI 2.378–10.488; p = 0.000) and manual labor occupation (OR 2.990; 95% CI 1.719–5.200; p = 0.000) (Table 9).

**TABLE 9:** Relationship between malnutrition (MNA) and level of physical activity (IPAQ-Short), according to demographic variables.

​	​	^a^Malnutrition (MNA)						
Demographic variables	Low or inactive physical activity	Yes	​	No	​	​	​	​
		n = 158	100%	n = 242	100%	OR	CI 95%	*p*
Age								
65–74	Yes	12	7.5	15	9.3	2.263	0.966–5.301	0.056
	No	35	21.7	99	61.5			
75–84	Yes	24	14.9	25	15.5	2.54	0.783–3.032	0.210
	No	43	26.7	69	42.9			
≥85	Yes	31	39.7	10	12.8	5.723	2.144–15.274	0.000
	No	13	16.7	24	30.8			
Sex								
Woman	Yes	43	17.8	25	10.4	2.413	1.353–4.302	0.002
	No	72	29.9	101	41.9			
Man	Yes	24	15.1	25	15.7	4.598	2.178–9.705	0.000
	No	19	11.9	91	57.2			
Marital status								
Single	Yes	41	21.1	28	14.4	2.125	1.168–3.865	0.013
	No	51	26.3	74	38.1			
Partnered	Yes	26	12.6	22	10.7	3.486	1.781–6.824	0.000
	No	40	19.4	118	57.3			
Educational level								
Low	Yes	61	19.5	35	11.2	2.655	1.616–4.363	0.000
	No	86	27.5	131	41.9			
High	Yes	6	6.9	15	17.2	4.88	1.311–18.166	0.012
	No	5	5.7	61	70.1			
Residence								
Rural	Yes	37	17.5	11	5.2	4.994	2.378–10.488	0.000
	No	66	31.1	98	46.2			
Urban	Yes	30	16.0	39	20.7	2.892	1.512–5.534	0.001
	No	25	13.3	94	50.0			
Job occupation								
Manual labor	Yes	53	20.1	26	9.8	2.99	1.719–5.200	0.000
	No	75	28.4	110	41.7			
Non-manual labor	Yes	14	10.3	24	17.6	2.99	1.279–6.990	0.010
	No	16	11.8	82	60.3			

^a^ Malnutrition corresponds to the sum of: malnutrition and risk of malnutrition.

## Discussion

Aging involves a process of both physiological and social changes that occur in the body over the years. Among these changes, eating patterns and nutritional status play a fundamental role, as inadequate nutrition can become a significant risk factor for morbidity and mortality, affecting the quality of life of this significant population (Velasco and Velásquez, 2023). Therefore, factors such as malnutrition, undernutrition, and obesity have negative consequences for the health of older adults (Encalada-Torres et al., 2022). However, adequate nutrition combined with regular physical activity benefits older adults in achieving and maintaining a healthy body weight (Moreno-Agostino et al., 2020), contributing to a decrease in the prevalence of NCDs.

### Demographic variables

In this context, the present study showed that the majority of the population were female, aged 65–84, with incomplete primary education and married. These results are similar to those obtained by Aquino et al. in Peru; who reported that the predominant older adults were women (54.1%) and married (Aquino et al., 2019). According to the level of education, the results were similar to the study conducted by Méndez Pérez et al. in Mexico, in which it was determined that 55% had a primary education; the average age being 71.3 ± 8.7 (Méndez-Pérez et al., 2021). Urban or rural residence is considered a demographic variable that influences the nutritional status of older adults. This study showed that 53% of older adults resided in a rural area, unlike the study by Vinuesa-Veloz et al., who found that the majority of older adults resided in urban areas (64.4% malnourished, 62.8% overweight, and 69% obese) (Vinueza et al., 2023).

### Nutritional status

Regarding BMI, this research found that normal nutritional status was most prevalent at 40.5%, followed by overweight at 30.8%, obesity at 18.5%, and thinness at 10.3%, findings similar to the study by Méndez Pérez et al. in Mexico. While, they were different from those obtained in the research carried out in Colombia by Molina where obesity with 6.8% represented the majority of the population, followed by underweight 23.8%, normal and overweight 7.7% each (Molina, 2019).

Regarding abdominal circumference, average values of 99.24 cm were observed in men and 95.25 cm in women. These values were similar to the average abdominal circumference obtained by Encalada-Torres et al., where the average was 100.55 (±7.41) cm in men and 96.36 (±10.19) cm in women (Encalada-Torres et al., 2019). Meanwhile, in the study by Durán et al., the average abdominal circumference was 94.2 cm and 85.1 cm in men and women over 75 years of age, which were lower than the present study (Durán et al., 2018).

The highest prevalence of malnutrition in this study was found in women with incomplete primary education, widows, and rural residents. These data are similar to those obtained by Pacurucu et al. and Vinueza et al., who established that malnutrition was more prevalent in women living in rural areas, associated with being widowed, being younger older adults, and having an educational level of 3.6 years (Pacurucu et al., 2019; Vinueza et al., 2023).

### Physical activity

The present study showed that moderate and low/inactive physical activity levels were the most frequent (43.3% and 29.3%, respectively); in the case of moderate physical activity, a higher concentration was evident in younger older adults, married women, and urban dwellers. This coincides with the results found by Vernaza et al. (Vernaza-Pinzón et al., 2017), who found that 55% of the population engages in moderate physical activity, with a higher prevalence among women. Meanwhile, the study by Vicentini D et al., conducted in Brazil, reported that 61% of older adults maintained an active or very active level of physical activity, presenting improved nutritional indicators (Vicentini et al., 2021).

### Mini Nutritional Assessment

Most older adults classified according to the MNA scale as having satisfactory nutritional status had a moderate level of physical activity according to the short IPAQ, followed by malnutrition with a low or inactive level of physical activity. These data are similar to those obtained in the study by Sugiura et al., who established that populations at high risk of malnutrition had difficulty performing physical activity (Lorés et al., 2020).

### Association between nutritional status (based on BMI) and physical activity

This study also observed a relationship between nutritional status based on BMI and low or inactive physical activity levels, highlighting malnutrition due to overweight. This result is similar to that obtained by Encalada-Torres et al., who found in their study that older adults are mostly obese and have moderate physical activity, which, in turn, is associated with hypertension (Encalada et al., 2018). For their part, Espinosa et al. determined in their study that older adults were malnourished, and this was associated, among other factors, with a sedentary lifestyle. They also highlighted malnutrition as a public health problem (Espinosa et al., 2019).

### Association between nutritional status according to MNA and physical activity

When analyzing the relationship between nutritional status based on MNA and low or inactive physical activity levels, it was found to be significant (p = 0.000), as in the study by Muñoz et al. in Colombia (p = 0.040). Establishing that older adults in the Comfamiliar group in Nariño had an unhealthy nutritional status and engaged in little physical activity, which influences their physical condition (Muñoz et al., 2020). In contrast, the present study differs from the results obtained by Chavarría et al., who found that obesity was linked to vigorous physical activity (Chavarría et al., 2017).

### Association between nutritional status according to BMI and demographic variables

According to the results obtained in this research, no factors were identified that were statistically significantly associated with malnutrition (BMI) and low or inactive physical activity level (IPAQ-Short), except in the case of older adults in manual labor occupations (p = 0.004); although it differs, in part, from what Otero & Rosas identified in relation to the fact that older women with higher education levels have a higher BMI. The present study and this research are related in terms of having a manual labor occupation that was associated, it did not show statistical significance (p > 0.05). For their part, Vanegas et al. obtained results that showed a high prevalence of malnutrition in older adults (74.5%) and a high percentage of physical inactivity (93.9%; p = 0.040), being higher in women (34.3%) (Vanegas et al., 2020).

Regarding the relationship between thinness (BMI) and low or inactive physical activity levels, this study did not demonstrate a statistical association; however, the majority of thin older adults were women aged 65–74 years, with a high educational level, low physical activity level, and urban residence. This data is consistent with Torres et al. (Torres et al., 2017), who demonstrated that low weight in the population studied was associated with age and educational level; while the results of the study by Lorés et al. found a statistically significant inverse relationship between physical activity and thinness by BMI (Lorés et al., 2020).

Regarding overweight (BMI) and low or inactive physical activity level (Short IPAQ), men with manual labor stood out as the main variables. These results are similar to those obtained by Gaona et al., who highlighted a higher prevalence of this condition in men (21.6%) than in women, with only 7.2% being overweight or obese (Gaona et al., 2018). This could be related to the findings of Hernández et al., who stated that men report higher alcohol consumption than women and tend not to exercise and smoke; this showed a negative relationship with BMI (Hernández et al., 2021).

In contrast to the findings of the study conducted by Barragán et al. Where it was shown that 77.41% and 22.58% of women and men, respectively, were obese according to BMI, in relation to low or inactive physical activity levels according to the IPAQ-Short (Barragán et al., 2018). In the present study, factors such as prevalence in men, low educational level, and manual labor were highlighted. This could be due to the fact that, as Barragán et al. points out, both income and educational level influence food selection and purchase, since people with a higher educational level tend to choose diets based on fruits, whole-grain bread, vegetables, and cereals, seeking to reduce their consumption of processed foods, sugar, and red meat. Furthermore, Molina found in her study a greater tendency among older men toward overweight (60.8%), also associated with their low physical activity and unhealthy eating habits (Molina, 2019).

A statistically significant association was demonstrated between malnutrition according to MNA and low or inactive physical activity level (Short IPAQ); this finding is related to that reported by Durán et al., which shows that physical activity was a protective factor for maintaining a healthy weight in older adults (Durán et al., 2018). In this sense, it is worth highlighting that, according to Giraldo et al., exercise helps preserve and improve the mobility and nutritional status of older adults, since it allows them to reduce body fat and reduce the occurrence of chronic non-communicable diseases such as diabetes and hypertension, among others (Giraldo et al., 2019).

Similarly, these findings are similar to a study conducted by Guerrero, who determined that 54.1% of the population was at risk of malnutrition and 21.2% were undernourished. He linked malnutrition with loss of muscle mass and functional limitations (Guerrero-García et al., 2016). While the findings of the study by Muñoz et al. did not show a statistically significant association between nutritional status and physical activity in older adults (p = 0.111), they did find an association between nutritional status and physical fitness (p = 0.04) (Muñoz et al., 2020). In contrast, Otero & Rosas found in their study a relationship between malnutrition, low educational attainment, and low socioeconomic status (Otero and Rosas, 2017). This suggests that malnutrition is a multidimensional problem associated with multiple factors, including physical activity.

As is generally known, regular physical activity contributes to maintaining muscle mass, improves basal metabolism, optimizes insulin sensitivity, and promotes energy balance; these are key factors in preventing overweight, obesity, and malnutrition in this age group. Furthermore, at a behavioral level, physically active older adults tend to exhibit better self-care patterns, functional mobility, and social participation, which positively impacts their nutritional status. Therefore, from a public health perspective, the findings of this research reinforce the need to promote community-based adapted physical activity programs, integrated into healthy aging strategies, to reduce the burden of chronic diseases, functional dependence, and healthcare costs associated with malnutrition.

However, the results should be interpreted considering certain limitations and potential confounding factors. Because this is a cross-sectional study, it is not possible to establish causality or the temporal direction of the observed association, as an altered nutritional status can also limit physical activity. Furthermore, variables such as advanced age, sex, and educational level can act as confounding factors if not adequately controlled, and the use of BMI in older adults may not accurately reflect body composition. The use of BMI in older adults may not accurately reflect body composition. Its significant limitations stem from its failure to differentiate between fat mass and muscle mass, often masking sarcopenia and fat distribution. An older adult with a normal BMI could have functional impairment, osteoporosis, or low lean mass. Therefore, higher cut-off points have been used, taking into account the WHO’s BMI guidelines for older adults. These considerations underscore the importance of longitudinal studies and incorporating more comprehensive measures to strengthen the evidence; however, it should be noted that such studies involve high research costs, which public institutions like ours often cannot afford.

## Conclusion

Although we are in the Decade of Healthy Aging (2021–2030), studies such as this one demonstrate that malnutrition, especially over nutrition, continues to rise and is associated with factors that could prevent it and reduce morbidity and mortality from non-communicable diseases (NCDs), such as physical activity. Therefore, physical activity is a key determinant of nutritional balance and overall health in older adults. The results of this research support the incorporation of nutritional assessment and physical activity level evaluation into primary healthcare programs and comprehensive geriatric assessments. Furthermore, the results allow us to recommend the implementation of integrated interventions that combine nutritional education, physical exercise adapted to older adults and their functional status, with an emphasis on community settings and senior care centers.

Likewise, the findings should guide the design and strengthening of public policies for active aging, promoting safe spaces for physical activity, training healthcare personnel, and allocating resources for preventive programs. Thus, this study provides relevant evidence for reducing malnutrition and preventing disability. This implies that all countries, and especially low-income countries such as those located in Latin America and the Caribbean, must commit to working with all social actors to generate or strengthen physical activity programs that improve the quality of life of older adults and their families.

## Data Availability

The original contributions presented in the study are included in the article/supplementary material, further inquiries can be directed to the corresponding author.
